# Supplementary material for: Organometallic compounds as carriers of extraterrestrial cyanide in primitive meteorites
Source: Nat Commun. 2019 Jun 25;10:2777. doi: 10.1038/s41467-019-10866-x (PMC6592946; doi:10.1038/s41467-019-10866-x)
Supplement: Supplementary file 1 — Supplementary Information [file 41467_2019_10866_MOESM1_ESM.pdf]

Supplementary Information

Organometallic compounds as carriers of extraterrestrial cyanide in primitive meteorites

Smith et al.

Supplementary Figures

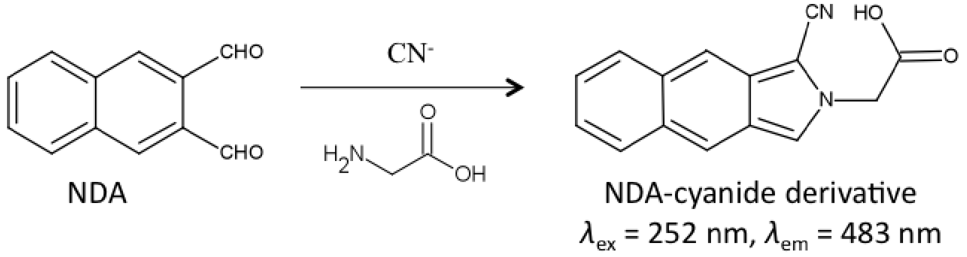

Supplementary Figure 1. Reaction scheme for the derivatization of cyanide anion. The NDA-cyanide derivative was measured in acid-digested distillates of various meteorites by LC-FD/ToF-MS to determine the amount of releasable cyanide. NDA is naphthalene-2,3-dicarboxaldehyde-cyanide.

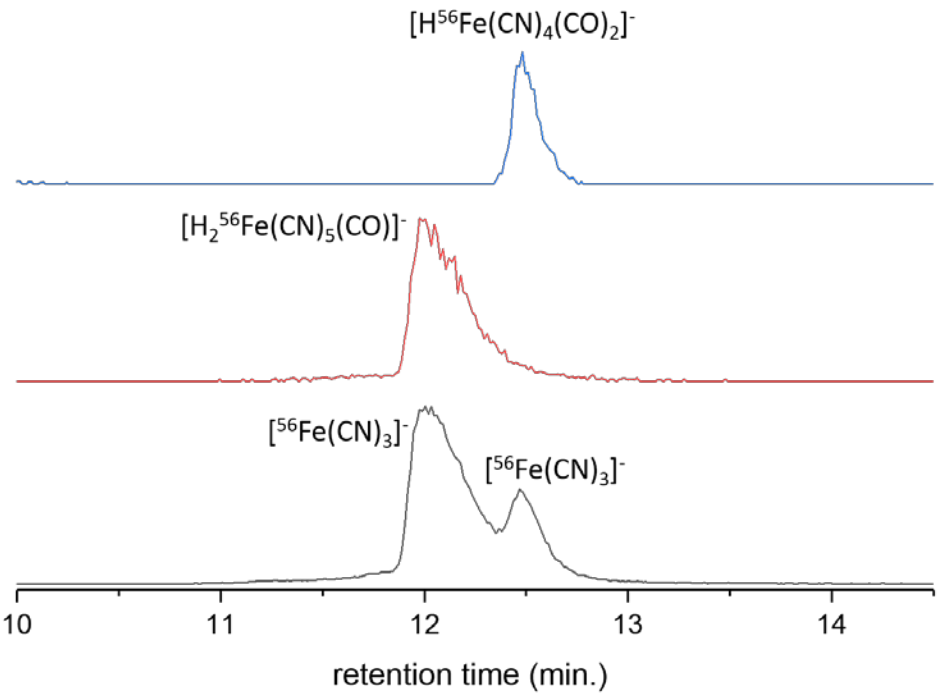

Supplementary Figure 2. Extracted ion chromatograms for LEW 85311 meteorite extract. Top blue trace, *m/z* = 216.9455, middle red trace, *m/z* = 215.9614, and bottom black trace, *m/z* = 133.9450 using a 5 ppm mass window. These data indicate that $[Fe(CN)_3]^-$ is a fragment ion common to both iron cyanocarbonyl complexes.

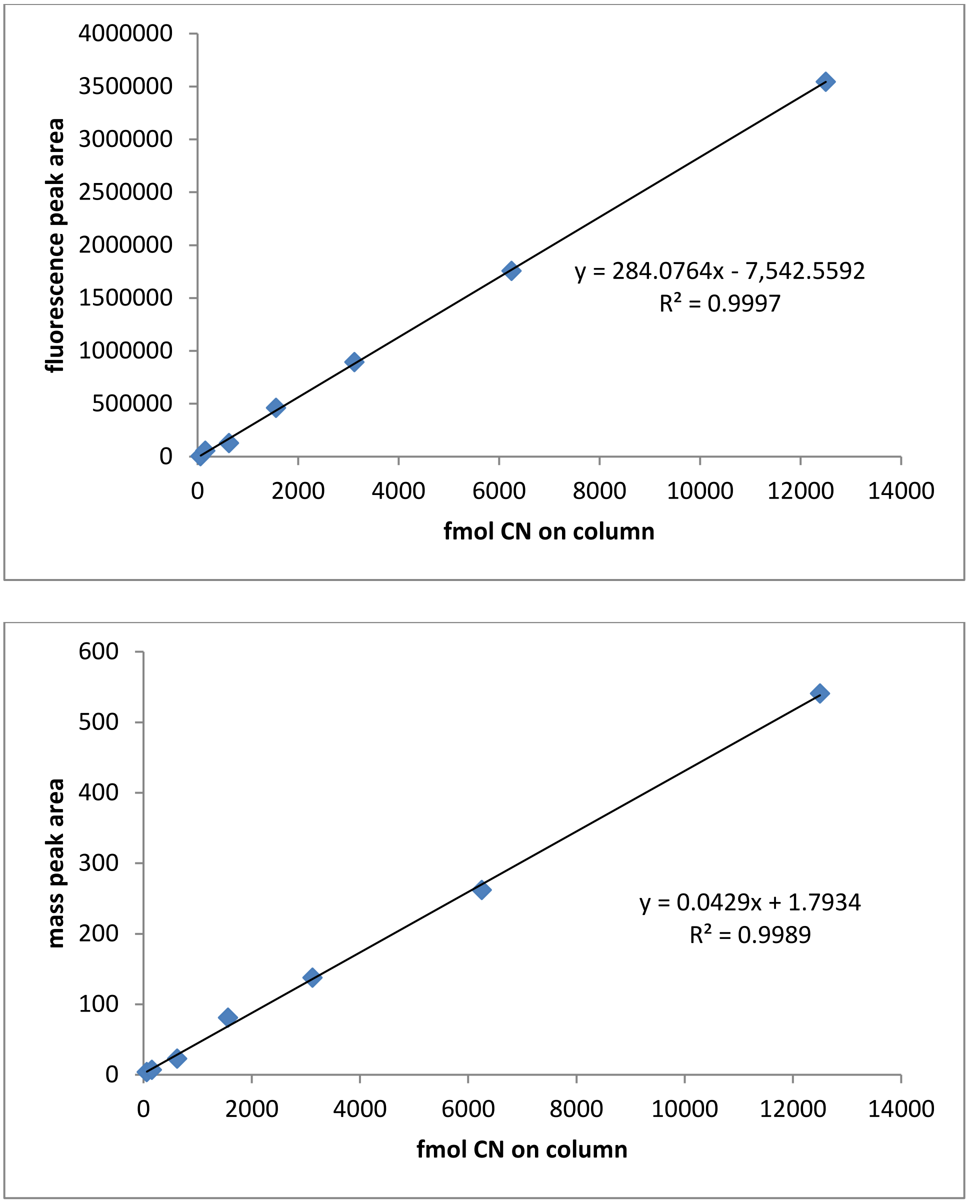

Supplementary Figure 3. Seven-point calibration curves. The fluorescence emission and single ion mass detection of the NDA-cyanide derivative (*m/z* 251.08 ± 0.03 Da; $[M+H]^+$) using non-distilled standard solutions of KCN are linear in the concentration range of 0.01 µM to 2 µM (or 62.5 fmol – 12.5 pmol on column). Source data are provided as a Source Data file.

Supplementary Tables

Supplementary Table 1. Element abundances in μmol·g^{-1} for LEW 85311 by ICP-MS

Element	H_2O extraction 100 °C for 23 h	0.08 M NH_4OH extraction 100 °C for 23 h
Na	189.8	172.7
Mg	359.1	181.0
Al	6.2	5.6
K	10.3	14.3
Ca	96.3	149.9
Ti	nd	nd
Fe	0.66	0.44
Co	nd	nd
Ni	0.17	0.24
Cu	nd	nd
Zn	nd	nd
Ag	0.17	0.07
Cd	nd	nd
Pt	0.01	0.01

nd = not detected in extract

Source data are provided as a Source Data file

Supplementary Notes

Supplementary Note 1: Method blanks.

Two method blanks were used in this study: a procedural blank and a serpentine blank. The procedural blank consisted of 500 µL 0.08 M NaOH added to 5.5 mL ultrapure water plus 750 µL 9 M H_2SO_4, which was distilled and derivatized using the same procedure as the meteorites. The procedural blank fluorescence and mass peak areas were subtracted from all standards and meteorite samples. The serpentine blank consisted of 203.6 mg serpentine (previously heated to 500 °C) added to 5.8 mL ultrapure water plus 750 µL 9 M H_2SO_4, which was distilled and derivatized using the same procedure as the meteorites. The serpentine blank is different from the procedural blank because it contains the meteorite mineral matrix, which rules out the possibility of making cyanide through mineral interactions during the acid-digestion and distillation procedure. The procedural blank and serpentine blank showed identical results.

Supplementary Note 2: Measurement of cyanide in non-CM and martian meteorites.

Cyanide was not detected above our detection limit (0.1 nmol $CN \cdot g^{-1}$ meteorite) in RBT 04133 (CV3) and GRA 06100 (CR2). It is possible that interferences, such as excessive sulfides and/or carbonates, which could have hindered the acid-digestion and distillation and/or derivatization processes, contributed to the non-detection; therefore, we also performed a spike experiment. We spiked 202.3 mg of RBT 04133 with 500 µL of 2 µM KCN, and processed this sample using the entire meteorite work-up procedure. This experiment resulted in a 27% recovery of the spike using the mass and fluorescence data.

This result demonstrated that if RBT 04133 had contained cyanide at a similar level to the CM carbonaceous chondrite with the lowest abundance of cyanide (ALH 83100 contained 49.8 ± 0.4 nmol $CN \cdot g^{-1}$ meteorite), the cyanide concentration would have been above our limit of detection, despite possible interferences and low spike recovery.

RBT 04133 is suggested to be a mildly thermally altered CV3 chondrite[1]. Additionally, Abreu et al. suggested that the CR parent body of the meteorite GRA 06100 may have had significant thermal alteration in addition to aqueous alteration[2]. Therefore, it is reasonable that thermal degradation of cyanide-containing species in the meteorite parent body may have been the cause for the lack of cyanide detection in these two meteorites.

Martian meteorites are igneous rocks that have been ejected from the surface of Mars by impacts. In the search for signatures of martian life or martian prebiotic chemistry, cyanide would be an excellent target because it plays a critical role in the abiotic synthesis of amino acids and nucleobases; however, cyanide was not detected above our detection limit in ALH 84001.

Supplementary Note 3: Study of HCN polymer.

Hydrogen cyanide and its polymer were made based on previously published methods[3-6]. First, HCN gas was produced by heating KCN and stearic acid under vacuum, then the gas was collected in a test tube cooled by a liquid nitrogen bath and double distilled under vacuum. HCN gas was then expanded into an evacuated glass bulb of known volume and frozen into a known mass of degassed (frozen-pumped-thawed) ultrapure water to a final concentration of 2 M. NH_3 gas was also frozen into ultrapure water in the same manner

to produce a 2 M solution. Once these solutions were thawed, they were combined to give a final solution of 1 M NH_4CN. The final NH_4CN solution was refluxed for 2 days during which an insoluble black solid formed. The solution was stored in a 50-mL centrifuge tube at room temperature with minimal headspace (air) and allowed to react (polymerize) for 2 years. The final color of the solution was dark brown with black solids.

Two aliquots of HCN polymer (~2 g each of combined liquid and black solid) were pipetted into tared MicroDIST distillation sample tubes (Lachat Instruments) and centrifuged under vacuum in a Centrivap (Labconco) overnight to a final dry weight of 20 mg each. 1 mL of 0.08 M NaOH was added to each sample tube with dry polymer, touch-mixed using a Vortex-Genie 2 (Scientific Industries), then centrifuged for 5 minutes. 500 μL of the supernatant was transferred to a separate vial and derivatized and analyzed using the same procedure as the meteorites in order to measure the free cyanide anion content of the polymer solution. 5.5 mL ultrapure water was added to the remaining 500 μL of polymer solution in the distillation sample tube. 750 μL 9 M H_2SO_4 was added to the polymer solution as a cyanide releasing agent and the full distillation tube was assembled. The HCN polymer sample was distilled for 30 minutes then derivatized with the NDA procedure the same as the meteorites and control samples.

The total cyanide concentration in the HCN polymer solution increased nearly 100-fold after distillation (from 0.8 nmol CN before to 79 nmol CN after distillation) indicating that cyanide can be released from HCN polymer by this method.

Supplementary Note 4: Study of acetonitrile and 3-cyanopyridine.

Organocyanides have been identified in the Tagish Lake meteorite (C2 ungrouped carbonaceous chondrite)[7]; however, these compounds are rarely reported in studies of meteoritic organic compounds (likely due to their susceptibility to hydrolysis). We investigated 3-cyanopyridine and acetonitrile, which served as model organocyanide compounds in meteorites, in order to see if these types of compounds could release cyanide using our experimental method.

3-cyanopyridine was acid-digested and distilled under the following conditions: 500 μL of 10 μM 3-cyanopyridine was added to 5.5 mL ultrapure water plus 750 μL 9 M H_2SO_4, which was acid-digested, distilled, and derivatized using the same procedure as the meteorites. In addition, non-distilled 3-cyanopyridine was analyzed for free cyanide anion in order to determine the free cyanide released after acid-digestion and distillation. Non-distilled 3-cyanopyridine was prepared as follows: 500 μL of 10 μM 3-cyanopyridine was added to 2 mL 0.08 M NaOH and derivatized using the same procedure as the meteorites. The 3-cyanopyridine did not release any cyanide after distillation (cyanide concentration was below limit of detection before and after distillation). Based on our results for 3-cyanopyridine, we hypothesize that cyano-substituted aromatic compounds (if present in CM chondrites) are unlikely to release appreciable amounts of cyanide using the acid-digestion and distillation procedure.

Acetonitrile (methyl cyanide) is the simplest organic nitrile and has been observed in the interstellar medium[8], in cometary comae[9, 10], and in the Murchison meteorite (as common pyrolysis products)[11, 12]. Therefore, acetonitrile can be considered a potential organonitrile in meteorites. Acetonitrile was distilled under the following conditions: 500

μL of acetonitrile was added to 5.5 mL ultrapure water plus 750 μL 9 M H_2SO_4, which was acid-digested, distilled, and derivatized using the same procedure as the meteorites. In addition, non-distilled acetonitrile was analyzed for free cyanide anion in order to determine the free cyanide released during acid-digestion and distillation. Non-distilled acetonitrile was prepared as follows: 500 μL of acetonitrile was added to 2 mL 0.08 M NaOH and derivatized using the same procedure as the meteorites. The total cyanide concentration in the acetonitrile increased 11-fold upon distillation (from 0.1 nmol free CN before to 1.1 nmol CN after distillation), which was less conversion than the HCN polymer. Therefore, it seems unlikely that acetonitrile and other simple alkyl nitriles are major sources for the detected cyanide in CM chondrites.

Supplementary Note 5: Free cyanide in LEW 90500.

Common aqueous and solid forms of cyanide are typically grouped into major classes such as free or simple cyanide (e.g., HCN, CN^-, KCN, NaCN), weak metal-cyanide complexes (dissociate under mildly acidic conditions), and strong metal-cyanide complexes (dissociate under strongly acidic conditions)[13]. To determine the amount of free CN in LEW 90500 (CM2 chondrite), we performed a mild extraction procedure, which did not utilize a strong acid or high temperature. A powdered sample of LEW 90500 (203.2 mg) was extracted using 2.5 mL 0.08 M NaOH (pH 12.45) and placed into a shaker at 25 °C for 17 hours. After settling, a small portion of this extract (50 μL) was then derivatized using our CN derivatization procedure and analyzed by LC-FD/ToF-MS method. Additionally, after shaking for 17 hours, a stir bar was added and the sample was stirred at room temperature overnight. The sample was centrifuged and 200 μL of

the supernatant was transferred to an HPLC vial and high-speed centrifuged again. A portion of this extract (50 μL) was then derivatized using our CN derivatization procedure and analyzed by LC-FD/ToF-MS method. The free CN was determined to be 10 - 11 nmol CN·g^{-1} LEW 90500, which corresponds to only ~7% of the total (acid-releasable) CN for LEW 90500 using either method (i.e. shaken and shaken and stirred). Therefore, we propose that additional sources must be present in LEW 90500 to explain the total (acid-releasable) cyanide and must be in another form other than free cyanide, such as metal-cyanide complexes (which was the case for LEW 85311).

Supplementary Note 6: Estimated concentration of iron cyanocarbonyls in LEW 85311. To estimate the abundance of iron cyanocarbonyl complexes in a 0.08 M NaOH extraction of LEW 85311, we used the peak areas from the two peak at 12 min. and 12.5 min. generated from the extracted ion chromatogram at *m/z* 133.9447 (bottom trace of Supplementary Figure 2) and compared them to the peak area generated from the extracted ion chromatogram at *m/z* 133.9447 of a 100 μM ferrocyanide standard solution. The peak at *m/z* 133.9447 is a $[Fe(CN)_3]^-$ fragment ion common to both iron cyanocarbonyl complexes as well as the ferrocyanide reference standard. We estimated the abundance of $[Fe^{II}(CN)_5(CO)]^{3-}$ and $[Fe^{II}(CN)_4(CO)_2]^{2-}$ at 266 and 98 nmol·g^{-1}, respectively, using the assumption that the signal response at *m/z* 133.9447 for ferrocyanide standard is identical to the signal response at *m/z* 133.9447 for each iron cyanocarbonyl complex at the same concentration. We also note the observation that cyanide content (est. 0.365 μmol·g^{-1} in the form of $[Fe^{II}(CN)_5(CO)]^{3-}$ and $[Fe^{II}(CN)_4(CO)_2]^{2-}$) seems to be an important contributing factor towards the amount of

solvent-extractable iron (ranging from 0.44 - 0.66 μmol·g^{-1} based on the extraction type). Next, if we assume complete dissociation of cyanide ligands from their iron centers, the releasable cyanide from $[Fe^{II}(CN)_5(CO)]^{3-}$ and $[Fe^{II}(CN)_4(CO)_2]^{2-}$ is calculated at 1332 and 393 nmol·g^{-1}, respectively, for a total releasable cyanide concentration of 1726 nmol CN·g^{-1}, which is about 70% of the acid releasable cyanide in LEW 85311 (2472 nmol CN·g^{-1}, which was reported in Table 1).

Supplementary Discussion

Using a range of cyanide abundances measured in the highest (LEW 85311) and lowest (ALH 83100) CM meteorites and an estimate of flux of carbonaceous chondrites[14] during the proposed Late Heavy Bombardment period of 8×10^{7} kg·year^{-1}, we estimate that the cyanide released from carbonaceous chondrites would be between 1×10^{2} and 5×10^{3} kg cyanide·year^{-1} (1.5×10^{1} to 7.4×10^{2} cyanide cm^{-2} s^{-1}, which was calculated from CN in the CM2 chondrite, nmol CN·g^{-1} × flux of carbonaceous chondrites during the proposed Late Heavy Bombardment, 8×10^{10} g·year^{-1} × Avogadro's number for a nanomole, 6.02×10^{14} molecules·nmol^{-1} × years per second, 3.17×10^{-8} year·s^{-1} / surface area of the Earth, 5.1×10^{18} cm^{2}). With an exogenous-favorable comparison, this abundance is about two to four orders of magnitude lower than the 7×10^{5} to 7×10^{7} kg cyanide·year^{-1} (1×10^{5} to 1×10^{7} cyanide cm^{-2} s^{-1}) estimated by endogenous syntheses on early Earth[15]. This suggests that meteorites may have acted as important point sources rather than a global source of HCN; however, these estimates should also be used cautiously as endogenous HCN synthesis can vary many orders of magnitude depending on the assumptions of the prebiotic atmosphere (such as CO_2 concentration and CH_4 mixing ratio)[15]. Furthermore, it is important to point out that our released cyanide abundance in CM chondrites represents values from modern analyses of meteorites. Dry radiolysis of organic compounds in meteorite parent bodies after the aqueous alteration period(s) would have continued due to long-lived radionuclides[16, 17], which has the potential to lower organic abundances over time. Cyanide abundances may not necessarily reflect those originally present ~4 Ga when these meteorites were delivered to early Earth; therefore, ~5000 kg cyanide·year^{-1} released from meteorites on early Earth may be an underestimate.

Therefore, we stress that any absolute comparison between estimated HCN from endogenous synthesis and estimated HCN from exogenous input should be approached cautiously.

Supplementary References

1. Davidson, J. et al. Petrography, stable isotope compositions, microRaman spectroscopy, and presolar components of Roberts Massif 04133: A reduced CV3 carbonaceous chondrite. *Meteorit. Planet. Sci.* **49**, 2133-2151 (2014).
2. Abreu, N.M. & Bullock, E.S. Opaque assemblages in CR2 Graves Nunataks (GRA) 06100 as indicators of shock-driven hydrothermal alteration in the CR chondrite parent body. *Meteorit. Planet. Sci.* **48**, 2406-2429 (2013).
3. Baronavski, A.P. Fluorescence-spectrum of HCN ($A \approx {}^1A'' \rightarrow X \approx {}^1\Sigma^+$) using ArF laser excitation. *Chem. Phys. Lett.* **61**, 532-537 (1979).
4. Gerakines, P.A., Moore, M.H. & Hudson, R.L. Ultraviolet photolysis and proton irradiation of astrophysical ice analogs containing hydrogen cyanide. *Icarus* **170**, 202-213 (2004).
5. Moore, M.H. & Hudson, R.L. Infrared study of ion-irradiated N_2-dominated ices relevant to Triton and Pluto: formation of HCN and HNC. *Icarus* **161**, 486-500 (2003).
6. de la Fuente, J.L., Ruiz-Bermejo, M., Nna-Mvondo, D. & Minard, R.D. Further progress into the thermal characterization of HCN polymers. *Polym. Degrad. Stabil.* **110**, 241-251 (2014).
7. Pizzarello, S. et al. The organic content of the Tagish Lake meteorite. *Science* **293**, 2236-2239 (2001).
8. Solomon, P.M., Jefferts, K.B., Penzias, A.A. & Wilson, R.W. Detection of millimeter emission lines from interstellar methyl cyanide. *Astrophys. J.* **168**, L107-L110 (1971).
9. Ulich, B.L. & Conklin, E.K. Detection of methyl cyanide in Comet Kohoutek. *Nature* **248**, 121-122 (1974).
10. A'Hearn, M.F. et al. Deep Impact: excavating Comet Tempel 1. *Science* **310**, 258-264 (2005).
11. Levy, R.L., Grayson, M.A. & Wolf, C.J. Organic analysis of Murchison meteorite. *Geochim. Cosmochim. Acta* **37**, 467-483 (1973).
12. Holzer, G. & Oro, J. Organic composition of the Allan Hills carbonaceous chondrite (77306) as determined by pyrolysis-gas chromatography-mass spectrometry and other methods. *J. Mol. Evol.* **13**, 265-270 (1979).
13. Dzombak, D. A., Ghosh, R. S. & Wong-Chong, G. M. Cyanide in Water and Soil Chemistry, Risk, and Management. (CRC Taylor & Francis, Boca Raton, FL, 2006).
14. Pasek, M. & Lauretta, D. Extraterrestrial flux of potentially prebiotic C, N, and P to the early Earth. *Orig. Life Evol. Biospheres* **38**, 5-21 (2008).
15. Tian, F., Kasting, J.F. & Zahnle, K. Revisiting HCN formation in Earth's early atmosphere. *Earth Planet. Sci. Lett.* **308**, 417-423 (2011).
16. Urey, H.C. The cosmic abundances of potassium, uranium, and thorium and the heat balances of the Earth, the Moon, and Mars. *Proc. Natl. Acad. Sci. U.S.A.* **41**, 127-144 (1955).
17. Urey, H.C. The cosmic abundances of potassium, uranium, and thorium and the heat balances of the Earth, the Moon, and Mars. *Proc. Natl. Acad. Sci. U.S.A.* **42**, 889-891 (1956).
